# Draft Genome Sequence of Zalerion maritima ATCC 34329, a (Micro)Plastic-Degrading Marine Fungus

**DOI:** 10.1128/mra.00017-23

**Published:** 2023-03-07

**Authors:** Ana Paço, Micael F. M. Gonçalves, João P. da Costa, Teresa A. P. Rocha-Santos, Artur Alves

**Affiliations:** a Department of Chemistry, University of Aveiro, Aveiro, Portugal; b Centre for Environmental and Marine Studies (CESAM), University of Aveiro, Aveiro, Portugal; c Department of Biology, University of Aveiro, Aveiro, Portugal; University of California, Riverside

## Abstract

Zalerion maritima is a marine fungus that has been studied for the biodegradation of (micro)plastics. Here, we report the draft genome sequence of strain ATCC 34329, which was shown to have a size of 58.4 Mb, a GC content of 44.39%, and 10,802 predicted genes.

## ANNOUNCEMENT

The marine fungus Zalerion maritima, from the family *Lulworthiaceae* (*Sordariomycetes*, *Ascomycota*), was defined in 1963 by Anastasiou (1). Since the 1970s, some studies have shown that *Z. maritima* has the ability to degrade different compounds, such as lignin (2), the insecticides aldrin and dieldrin (3), polyurethane (4), and polyethylene (5). The latter two compounds, plastics materials, indicate that this fungus could be an interesting agent for application in the biodegradation of plastics, specifically plastics present in the marine environment. So, to increase our knowledge of the metabolic pathways and mechanisms involved in biodegradation (6), we sequenced and annotated the genome of *Zalerion maritima* (ATCC 34329), obtained in 2017 from the ATCC and maintained in our lab since then.

Using the guanidinium thiocyanate method (7), genomic DNA was extracted from mycelium that had been cultured in potato dextrose broth at 25°C for 7 days. The quality of the DNA was checked using 0.8% agarose gel electrophoresis, and the concentration was determined using the NanoDrop 2000 spectrophotometer (Thermo Fisher Scientific Inc., Waltham, MA, USA). A genome sequence was obtained using the Illumina NovaSeq 6000 XP system in paired-end 150-bp (PE150) format with an S2 flow cell; the libraries were constructed by Eurofins (Brussels, Belgium) by “combining 3–4 paired-end shot gun libraries with 2 long jumping distance library [*sic*]” (https://www.eurofins.in/genomics/next-generation-sequencing/genome-sequencing/).

The quality of the raw sequence data generated was assessed using the program FastQC (8), and assembly was conducted using SPAdes v.3.14 (9) with default parameters. Subsequently, the nuclear genome was analyzed using the Web application QUAST (10) to assess the quality of the assembled genome. The genome has a total length of 58.4 Mb assembled in 2,208 contigs, the largest of which comprises 455,379 bp. The other genome statistics are presented in Table 1. Gene prediction was performed using WebAUGUSTUS (11) with default parameters, trained (12) on the optimized algorithm for Xylaria grammica. The Web-based application dbCAN (13) was used to predict carbohydrate-degrading enzymes (CAZymes), with default settings. Among the genes encoding CAZymes, some encoded auxiliary activities/oxidoreductases, such as laccase, an enzyme associated with biodegradation of plastics.

**TABLE 1:** Statistics for *Zalerion maritima* genome assembly and annotation

Characteristic	Value
Total assembly length (bp)	58,434,198
No. of contigs (≥500 bp)	2,208
%GC	44.39
*N*_50_ (bp)	66,184
BUSCO completeness (%)	98.35
Sequencing depth (×)	126
No. of genes predicted using AUGUSTUS	10,802
No. of CAZymes	491
No. of regions identified using antiSMASH	1,823
No. of antiSMASH biosynthetic gene clusters	12
No. of annotated genes against the NR database^*a*^	8,858
No. of functional annotated genes against KEGG	677
No. of genes	
Assigned using GO^*b*^ terms	4,932
Assigned using InterPro	9,875
Assigned and predicted using EggNOG	7,563

a NR, nonredundant.

b GO, Gene Ontology.

The Web-based application antiSMASH v.5.0 (14) was used to screen for the presence of biosynthetic gene clusters involved in secondary metabolite synthesis, using the “relaxed” strictness option.

The software OmicsBox v.1.4.12 was used to functionally annotate the predicted genes using Blast2GO against NCBI’s nonredundant (NR) protein database, the Kyoto Encyclopedia of Genes and Genomes (KEGG), and the Gene Ontology (GO) knowledgebase with an E value threshold of 1 × 10^−3^. InterProScan and EggNOG (Evolutionary Genealogy of Genes: Non-supervised Orthologous Groups) were used to classify the proteins with an E value of 1 × 10^−3^. The annotation results obtained using CAZymes, antiSMASH, and Blast2GO are presented in Table 1. By comparison against the NR database and using EggNOG, proteins already associated with biodegradation of plastics were identified, such as laccase and its precursor, monooxygenase, cutinase, and cytochrome P450, among others (15–18). KEGG analysis revealed proteins involved in 115 different pathways, some of them related to the degradation of chemical compounds, such as the atrazine degradation pathway or the citrate cycle (TCA cycle) (16, 19).

This work will serve as an important resource for further studies regarding the ability of fungi to use plastic as a carbon source. This genomic characterization will also provide insights for future genomic analyses of marine fungi and contribute to unraveling aspects of marine fungal biology and ecology.

### Data availability.

This whole-genome shotgun project has been deposited at DDBJ/ENA/GenBank under the accession number JAKWBI000000000. The version described in this paper is version JAKWBI020000000. The raw sequence reads were deposited in the SRA under the accession numbers SRR18275426 and SRR18275427.
